# Role of aromatase inhibitors in breast cancer

**DOI:** 10.1038/sj.bjc.6602688

**Published:** 2005-08-15

**Authors:** R Carpenter, W R Miller

**Affiliations:** 1 The Breast & Endocrine Unit, 2nd Floor West Wing, St Bartholomew's Hospital, London EC1A 7BE, UK; 2 Edinburgh Breast Unit Research Group, Western General Hospital, University of Edinburgh, Paderewski Building, Edinburgh EH4 2XU, UK

**Keywords:** aromatase inhibitors, tamoxifen, breast cancer, future treatment

## Abstract

Primarily, the role of the aromatase inhibitors has been investigated in postmenopausal women with breast cancer, although it is also now being assessed in premenopausal patients following ovarian ablation/suppression. Aromatase inhibitors markedly suppress endogenous oestrogens without directly interacting with oestrogen receptors, and thus have a different mechanism of action to the antioestrogen, tamoxifen. The inhibitors may be divided into subgroups according to their structure (steroidal and nonsteroidal), and there appears to be a lack of cross-resistance between the classes of aromatase inhibitors enabling them to be used sequentially and potentially to prolong endocrine hormone therapy. In addition, with increased efficacy and favourable safety and tolerability profiles, the aromatase inhibitors are starting to challenge tamoxifen as first choice endocrine treatment in a number of settings. Potential differences in side-effect profiles may appear between the steroidal and nonsteroidal aromatase inhibitors when used in long-term settings. Thus, it has been suggested that the steroidal agents have favourable end organ effects; for example, the steroidal inhibitor, exemestane, has minimal negative effects on bone and lipid metabolism in animal and clinical studies. This paper provides an overview of the current and future roles of aromatase inhibitors for breast cancer treatment.

With many breast cancers requiring oestrogen for growth, hormone therapy has focused on suppressing the synthesis and action of these hormones. The gold standard for hormone therapy in premenopausal women has been ovarian ablation, with antioestrogens (such as tamoxifen) being primarily used in postmenopausal patients. However, in postmenopausal women, an alternative approach is to target the aromatase enzyme, which is responsible for the conversion of androgens to oestrogens and is the key step in oestrogen biosynthesis. Recent clinical trial results with aromatase inhibitors suggest this approach has an important role in treatment of ER and/or PgR positive breast cancers.

## RATIONALE FOR AROMATASE INHIBITORS IN BREAST CANCER

Oestrogen deprivation has long been an effective treatment for breast cancer (Beatson, 1896), and the introduction of the antioestrogen, tamoxifen, in the 1970s produced marked improvements in breast cancer survival (EBCTCG, 1998). Tamoxifen blocks the oestrogen receptor, whose signalling represents a critical growth and survival pathway in breast tumours. However, tamoxifen also has some partial oestrogen agonist effects, which may be detrimental, and may be associated with increased risk of uterine cancer and thromboembolism. Agonist activity may also be involved in the development of tamoxifen resistance (Smith and Dowsett, 2003).

An alternative approach to blocking the oestrogen receptor is to reduce levels of its ligand, oestradiol, by targeting aromatase. This enzyme converts androstenedione and testosterone to oestrone and oestradiol (Gruber *et al*, 2002). It is a member of the cytochrome *P*450 class and is highly expressed in the placenta and in the granulosa cells of ovarian follicles. However, it is also present in peripheral tissues, including adipose tissue, liver, muscle, brain and breast cancer tissue (Miller, 1991; Simpson and Davis, 2001).

**Figure 1:**
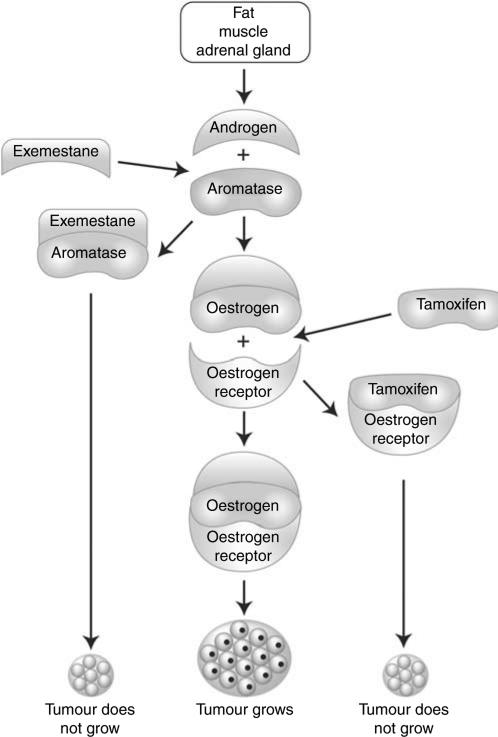
Mechanisms of action of tamoxifen and exemestane.

Unlike tamoxifen, aromatase inhibitors do not directly interact with the oestrogen receptor but indirectly affect signalling in postmenopausal women by blocking the conversion of adrenal androgens to oestrogen in peripheral (ie non-ovarian) tissues, including the breast itself (Figure 1; Miller, 1991). Enhanced aromatase activity has been observed in adipose tissue adjacent to breast cancers (O’Neill *et al*, 1988), and may promote growth of malignant cells (O’Neill *et al*, 1988). Aromatase inhibitors inhibit *in situ* oestrogen synthesis in both tumour and non-malignant breast tissue (Miller and Dixon, 2001), and have demonstrated efficacy in suppressing peripheral oestrogen synthesis in postmenopausal women (Smith and Dowsett, 2003).

Aromatase inhibitors may be divided into two subtypes, steroidal and nonsteroidal. Steroidal inhibitors (eg exemestane) are analogues of androstenedione and bind irreversibly to the substrate binding site on the aromatase molecule, and are also known as enzyme inactivators. Nonsteroidal inhibitors (eg anastrozole and letrozole) bind reversibly to the haem group of the enzyme. The clinical significance of this difference in mechanism of action is uncertain at present, although it may enable the different types of agent to be used sequentially in order to prolong clinical response (Lønning *et al*, 2000; Bertelli *et al*, 2002). Later in the supplement, Dr Bertelli discusses sequencing of therapy further.

Several trials have shown that aromatase inhibitors are effective for the treatment of postmenopausal women who have experienced disease progression while on tamoxifen and in the first-line setting (Nabholtz *et al*, 2000; Milla-Santos *et al*, 2000; Dirix *et al*, 2001; Mouridsen *et al*, 2001). In the adjuvant and neoadjuvant settings:

- The Arimidex, Tamoxifen Alone or in Combination (ATAC) study (ATAC, 2002; Buzdar, 2004) has shown the aromatase inhibitor, anastrozole, to be more effective than tamoxifen in terms of disease-free survival (DFS) with several important tolerability benefits.
- The Intergroup Exemestane Study (IES) showed that switching to exemestane after only 2–3 years of tamoxifen therapy significantly improved DFS *vs* the standard 5 years of tamoxifen therapy (Coombes *et al*, 2004).
- Results from the MA-17 trial indicated that following the completion of standard adjuvant tamoxifen therapy, letrozole treatment improved DFS compared with placebo (Goss *et al*, 2003).
- In the neoadjuvant setting, the P024 trial demonstrated that women with oestrogen receptor-positive primary breast cancer who received letrozole were more likely to receive breast conserving surgery compared with those treated with tamoxifen (Ellis *et al*, 2001).

## CURRENT GUIDELINES AND THE AROMATASE INHIBITORS

Both the National Comprehensive Cancer Network (2005) and The Cancer Care Ontario Practice Guidelines Initiative (Breast Cancer Disease Site Group, 2002) have added aromatase inhibitors to their guidelines alongside tamoxifen as first-line therapy in postmenopausal oestrogen receptor-positive women with advanced breast cancer. The European Society of Mastology has also added aromatase inhibitors as first-line therapy in their advanced breast cancer guidelines (Blamey, 2002).

Following the publication of the first results from the ATAC study (ATAC, 2002), the American Society of Clinical Oncology (ASCO) reviewed their guidelines on adjuvant treatment of breast cancer (Winer *et al*, 2002, 2003). Their recommendations were that a 5-year course of tamoxifen should remain as standard therapy with adjuvant therapy with an aromatase inhibitor only in postmenopausal women with a relative or absolute contraindication to tamoxifen.

## CURRENT PRACTICE

In advanced disease, in the first-line setting, anastrozole, letrozole and exemestane have proved to be more clinically efficient than tamoxifen in prospective randomised trials and demonstrate a more favourable side-effect profile (Bonneterre *et al*, 2000; Nabholtz *et al*, 2000; Paridaens *et al*, 2000, 2003; Mouridsen *et al*, 2001; Howell *et al*, 2003). In previous studies, all three have also demonstrated superiority over megestrol acetate in the second-line setting after tamoxifen failure (Buzdar *et al*, 1996, 1997; Dombernowsky *et al*, 1998; Goss *et al*, 1999; Kaufmann *et al*, 2000). Exemestane and letrozole have been shown to be active and well tolerated after tamoxifen and megestrol acetate failure in the third-line setting (Ingle *et al*, 1997; Jones *et al*, 1999). Furthermore, an apparent lack of cross-resistance between the aromatase inhibitors offers the possibility of sequential use in advanced disease (see Dr Bertelli's article later in this supplement).

In the neoadjuvant setting, letrozole has proved to be superior to tamoxifen both in terms of tumour response and breast conservation rate (Eiermann *et al*, 2001). Interestingly, a biological evaluation of the study showed that tumours which overexpressed HER2 receptors were more likely to respond to the aromatase inhibitor than to tamoxifen (Ellis *et al*, 2001). Both anastrozole and exemestane have also been shown to be more effective than tamoxifen in the preoperative setting (Dixon *et al*, 1999a, 1999b; Geisler *et al*, 1999; Miller and Dixon, 2002).

In the adjuvant setting, while tamoxifen is the current standard of care, four large randomised controlled studies have favourably evaluated aromatase inhibitors against tamoxifen either ‘head to head’ or in sequence with adjuvant tamoxifen (ATAC, 2002; Goss *et al*, 2003; Buzdar, 2004; Coombes *et al*, 2004; Jakesz *et al*, 2004). Interestingly, an analysis of the ATAC data shows that ER +ve, PR −ve patients may particularly benefit from an aromatase inhibitor (ATAC, 2002; Buzdar, 2004).

## SAFETY ISSUES

Data from postmenopausal women with advanced disease suggest that steroidal and non-steroidal aromatase inhibitors have similar tolerability profiles. In the ATAC study, patients receiving anastrozole had fewer vascular and uterine adverse events than those receiving tamoxifen (ATAC, 2002; Buzdar, 2004). Exemestane was associated with less severe flushing, sweating, nausea and oedema than tamoxifen (Paridaens *et al*, 2000; 2003). The most commonly reported adverse events in women treated with aromatase inhibitors were hot flushes, nausea, vomiting, headache and fatigue.

Emerging data suggest that there may be differences between the two types of aromatase inhibitor in their effects on end organs. The steroidal agent exemestane may have positive or neutral effects on lipid metabolism, as opposed to nonsteroidal agents which have shown unfavourable or neutral effects, and may have less impact on bone turnover than the nonsteroidals (Martinetti *et al*, 2003). Studies with exemestane have shown mixed effects on bone; exemestane has been seen to reverse the increased bone turnover induced by ovariectomy in an animal model (Goss *et al*, 2001), and in a comparison with letrozole, exemestane reduced bone resorption markers (Goss *et al*, 2002). However, other studies have also shown increased levels of bone turnover markers with exemestane (Martinetti *et al*, 2003; Geisler *et al*, 2004). In the adjuvant setting, increased osteoporosis has been demonstrated in trials with all the third-generation aromatase inhibitors (ATAC, IES, ARNO, MA17). Any differences between the aromatase inhibitors will become evident with long-term use (eg in adjuvant or prevention settings). Clinical trials are currently under way to investigate these effects. The consequences of the aromatase inhibitors on bone and lipids are explored further later in the supplement.

## FUTURE ROLE FOR AROMATASE INHIBITORS

### Advanced disease

Anastrozole and letrozole are already licensed for first-line therapy in advanced disease. Evidence suggests that in future exemestane may also be approved for first-line use. Results from a phase II, randomised, EORTC study comparing exemestane with tamoxifen treatment in patients who had previously received no hormonal therapy for metastatic disease revealed a better overall response rate for exemestane than tamoxifen (41 *vs* 17%) (Paridaens *et al*, 2003). There was a lower incidence of severe flushing, sweating, nausea and oedema in women who received exemestane than tamoxifen. This study was extended into a phase III trial comparing time-to-disease progression of the two agents, and results for 382 patients were presented at the 2004 ASCO meeting. Median progression-free survival was found to be significantly longer with exemestane than tamoxifen (10.9 *vs* 6.7 months, *P*=0.04) and safety/tolerability assessments were also favourable for the aromatase inhibitor (Paridaens *et al*, 2004).

### Neoadjuvant therapy

Currently, letrozole is the only aromatase inhibitor licensed for use as neoadjuvant therapy, based on the results of the P024 trial (Ellis *et al*, 2001), where more women with oestrogen receptor-positive (ER+ve) primary breast cancer who received letrozole went on to receive breast conserving surgery (60% responded and 48% underwent successful breast-conserving surgery) compared with tamoxifen (41% responded and 36% underwent breast conservation). Several other studies have suggested that aromatase inhibitors may be more effective than tamoxifen in reducing tumour size prior to surgery (Dixon *et al*, 1999a, 1999b; Geisler *et al*, 1999; Miller and Dixon, 2002).

### Adjuvant therapy

Tamoxifen is the most widely used endocrine agent for adjuvant therapy, but it has drawbacks, in particular an increased risk of thromboembolic events and endometrial changes, including endometrial cancer (EBCTCG, 1998; Baum, 1999). Adjuvant trials of all third generation aromatase inhibitors show superior efficacy and equivalent or better tolerability. However, there are several difficulties in assessing the role of adjuvant endocrine therapy. Endocrine therapy is often administered in consort with chemotherapy making it difficult to separate the endocrine effects of the two types of treatment. Further head-to-head and sequencing trials are currently running that should add to our knowledge regarding use of the aromatase inhibitors in these settings. As these trials report, it is hoped that a consensus will emerge regarding whether aromatase inhibitors may replace tamoxifen in the adjuvant setting or whether sequencing with tamoxifen is more appropriate. Trials include:

*TEAM*: comparing adjuvant exemestane *vs* adjuvant tamoxifen for 5 years in 4400 postmenopausal women with early breast cancer. This is a phase III randomised trial.

*BIGFEMTA*: comparing 5 years of tamoxifen, 5 years of letrozole, or sequenced therapy of 2–3 years each starting with either tamoxifen or letrozole. The trial recruited a total of 8028 patients between 1998 and 2003. Primary Core Analysis results presented at St Gallen 2005 show that letrozole significantly increased DFS *vs* tamoxifen (*P*=0.003) at a median follow-up of 35.5 months (Thürlimann, 2005).

*ARNO*: comparing 3 years’ treatment of anastrozole with 3 years of tamoxifen, following 2 years’ treatment with tamoxifen. The trial began in 1996, with initial data recently being reported in combination with results from the ABCSG Trial (total *n*=3123; Jakesz *et al*, 2004). After a median follow-up of 26 months, the hazard ratio for recurrence-free survival was 0.59 for anastrozole *vs* tamoxifen (95% CI=0.42–0.82; *P*<0.0018), and 0.61 for distant recurrence-free survival (95% CI 0.42–0.87; *P*=0.0067).

*ICCG*: comparing 2 years’ treatment of exemestane with 2 years of tamoxifen, following 3 years’ treatment with tamoxifen.

### Unresolved issues

Evidence from the ATAC trial suggests that combining tamoxifen with anastrozole does not give additional benefit with results equivalent to tamoxifen alone and worse than anastrozole alone. The likely explanation for this is that in the low oestrogen environment created by an aromatase inhibitor, tamoxifen is more likely to display some agonist activity. Further, there is evidence in the advanced setting that use of a steroidal aromatase inhibitor after a nonsteroidal agent or *vice versa* can prolong clinical benefit (Lønning *et al*, 2000; Bertelli *et al*, 2002). Therefore, more work is needed to determine the optimal sequence of tamoxifen, steroidal and nonsteroidal aromatase inhibitors.

### Disease prevention

Significant reduction in the emergence of contralateral breast cancer in all current adjuvant studies of third generation aromatase inhibitors raises the question of whether they might have a role in disease prevention, particularly bearing in mind their favourable safety/tolerability profile, an especially important consideration in this setting. Oestrogen has been implicated in the development of breast cancer, and oestrogen metabolites may also be directly carcinogenic; therefore, reduction in oestrogen levels in high-risk women with high levels of oestrogen may be beneficial. Among ongoing prevention trials are:

*IBIS II:* aims to determine the effectiveness of anastrozole *vs* placebo in preventing breast cancer in healthy postmenopausal women at risk, and comparison between tamoxifen and anastrozole in postmenopausal women with ductal carcinoma *in situ* in 10 000 women.

*ApreS:* compares exemestane with placebo in prevention of breast cancer in 666 women with the BRACA 1/2 mutation (Bevilacqua *et al*, 2001).

### Premenopausal women

Another possible role for aromatase inhibitors may be following ovarian ablation/suppression in premenopausal women. Three important, linked trials are investigating this:

*SOFT*: examining tamoxifen alone *vs* tamoxifen plus ovarian function suppression *vs* exemestane plus ovarian function suppression in 3000 patients.

*TEXT*: investigating the use of tamoxifen plus a gonadotropin-releasing hormone (GnRH) analogue with or without chemotherapy *vs* exemestane plus a GnRH analogue with or without chemotherapy in 1845 patients.

*PERCHE*: randomising patients from the TEXT trial into PERCHE if the investigator and/or patient are uncertain about the role of chemotherapy added to the complete oestrogen blockade – the choice of whether or not to use chemotherapy can be determined by randomisation in the PERCHE trial. Patients can be enrolled in both trials – first PERCHE (to determine whether or not chemotherapy is used) and then TEXT (to determine choice of tamoxifen or exemestane). PERCHE aims to enroll 1750 patients.

### Combination with cyclooxygenase 2 (COX2) inhibitors

Following observations of cyclooxygenase 2 (COX2) involvement in breast carcinogenesis, the therapeutic potential of combining aromatase inhibitors with COX2 inhibitors is currently being investigated. Studies have suggested that this might bring improved response (Pesenti *et al*, 2001; Canney, 2003; Dirix *et al*, 2003). Early results of a feasibility study of exemestane plus the specific COX2 inhibitor celecoxib in postmenopausal women with histologically proven advanced or metastatic breast cancer showed 31% with a partial response and 42% having stable disease following treatment with both agents (Canney, 2003). Now the following clinical trials are underway:

- NCIC CTG MA 27 is a randomised 2 × 2 trial comparing exemestane and anastrozole with or without celecoxib in 6800 women.
- The International Cooperative Breast Cancer Group/Breast International Group (ICCG/BIG) trial is a phase III study looking at sequential exemestane and tamoxifen with celecoxib *vs* placebo.

Professor Bundred gives further consideration to this potential combination later in this supplement.

## CONCLUSIONS

Aromatase inhibitors are rapidly proving to be a valuable addition to breast cancer therapy in postmenopausal women with oestrogen receptor positive tumours. As results of ongoing clinical trials become available, it is likely that these agents will be used earlier in the course of the disease and in the prevention setting, and may be used sequentially to prolong hormonal therapy and delay the need for chemotherapy, and possibly become part of a combination approach with other agents such as the COX2 inhibitors.
